# Fetal intracerebral hemorrhage: review of the literature and practice considerations

**DOI:** 10.1038/s41390-025-04000-5

**Published:** 2025-03-18

**Authors:** Mary Dunbar, Sonika Agarwal, Charu Venkatesan, Brigitte Vollmer, Barbara Scelsa, Andrea C. Pardo, Tomo Tarui, Anthony R. Hart, Sarah B. Mulkey, Monica E. Lemmon, Dawn Gano

**Affiliations:** 1 Department of Pediatrics, Hotchkiss Brain Institute, University of Calgary, Calgary, AB Canada; 2 Division of Neurology & Pediatrics, Children’s Hospital of Philadelphia; Division of Neurology, Perelman School of Medicine at the University of Pennsylvania, Philadelphia, PA USA; 3 Division of Neurology, Department of Pediatrics, Cincinnati Children’s Hospital Medical Center, University of Cincinnati College of Medicine, Cincinnati, OH USA; 4 Clinical Neurosciences, Clinical and Experimental Sciences, Faculty of Medicine, University of Southampton; Paediatric and Neonatal Neurology, Southampton Children’s Hospital, University Hospital Southampton NHS Foundation Trust, Southampton, UK; 5 Department of Pediatric Neurology, Buzzi Children’s Hospital, University of Milan, Milan, Italy; 6 Department of Pediatrics, Division of Neurology, Ann & Robert H. Lurie Children’s Hospital of Chicago, Northwestern University Feinberg School of Medicine, Chicago, IL USA; 7 Division of Pediatric Neurology, Hasbro Children’s Hospital, Warren Alpert Medical School of Brown University, Providence, RI USA; 8 Department of Paediatric Neurology, King’s College Hospital NHS Foundation Trust, London, UK; 9 Departments of Neurology and Pediatrics, The George Washington University School of Medicine and Health Sciences, Washington, DC USA; 10 Zickler Family Prenatal Pediatrics Institute, Children’s National Hospital, Washington, DC USA; 11 Department of Pediatrics and Population Health Sciences, Duke University School of Medicine, Durham, NC England; 12 Department of Neurology and Pediatrics, University of California San Francisco, San Francisco, CA USA

## Abstract

**Abstract:**

Fetal intracerebral hemorrhage is increasingly recognized on prenatal imaging. In this review, we discuss clinically relevant aspects of fetal intracerebral hemorrhage, including germinal matrix-intraventricular hemorrhage, as well as intraparenchymal hemorrhage. We discuss current clinical practice for prenatal counseling and postnatal management of fetal intracerebral hemorrhage, and offer practical recommendations for clinicians. We propose standardized terminology for classification of fetal intracerebral hemorrhage to be used in future research. We also highlight gaps in the literature and priorities for future research, namely the need for prospective large-scale studies to better understand underlying etiologies and neurodevelopmental outcomes in fetal intracerebral hemorrhage.

**Impact statement:**

- We discuss the diverse etiologies and outcomes of fetal intracerebral hemorrhage, and propose standardized terminology for classification.
- We outline current practice and offer practical recommendations for management and counseling of fetal intracerebral hemorrhage, recognizing the need for capacity-building in the newly emerging subspecialty of fetal neurology.
- We highlight gaps in the literature and research priorities in fetal intracerebral hemorrhage to promote collaborative research, and the development of interventions to improve pregnancy and child outcomes.

## Introduction

Intracranial hemorrhage (ICH) is increasingly diagnosed prenatally, in part due to the increasing availability and use of advanced prenatal diagnostic imaging.^1^ The timing of diagnosis of fetal ICH depends on the timing of hemorrhage, timing of imaging, indication for imaging, type of imaging, and whether there are conditions such as maternal trauma or illness. Often, the discovery of fetal ICH may be incidental.^2^ Fetal ICH can occur in different compartments, including epidural, subdural, subarachnoid, subpial, intraparenchymal, and intraventricular.^3,4^

**Fig. 1:**
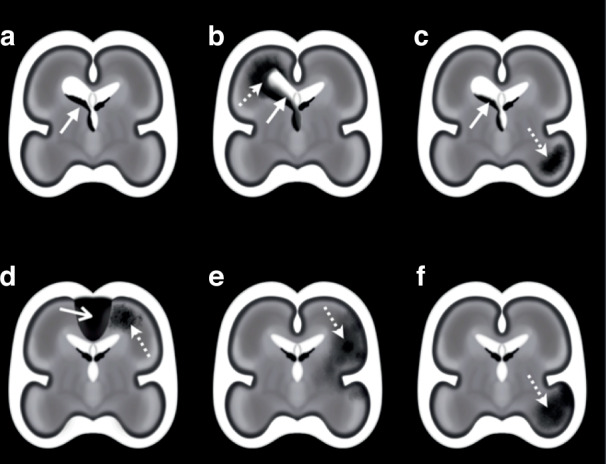
Fetal intracerebral hemorrhage discussed in this guideline. **a**–**c** Intraventricular Hemorrhage (IVH); **d**–**f** Intraparenchymal Hemorrhage (IPH) without intraventricular hemorrhage. **a** Isolated intraventricular hemorrhage such as germinal matrix hemorrhage (GMH-IVH; arrow). **b** Intraventricular hemorrhage (solid arrow) with subsequent periventricular hemorrhagic infarction (PVHI; dotted arrow). **c** Intraventricular hemorrhage (solid arrow) with separate area of intraparenchymal hemorrhage (dotted arrow). **d** Intraparenchymal hemorrhage (dotted arrow) associated with a cerebral sinovenous thrombosis (CSVT), also known as a dural venous sinus thrombosis (DVST; solid open arrow). **e** Intraparenchymal hemorrhage (dotted arrow) in an arterial distribution suspicious for hemorrhagic transformation of an arterial infarct (Fetal Arterial Ischemic Stroke; FAIS). **f** intraparenchymal hemorrhage not associated with IVH, clot, nor consistent with hemorrhagic transformation of an arterial infarct suspected to be an idiopathic hemorrhage (Fetal Hemorrhagic Stroke; FHS). Illustrations are meant to reflect anatomy at ~25 gestational weeks. Refer to Fig. 2 for the corresponding proposed classification flowchart.

In this review, we will cover clinically relevant aspects of fetal intracerebral hemorrhage, specifically intraparenchymal hemorrhage (IPH), as well as germinal matrix-intraventricular hemorrhage (GMH-IVH) and its sequelae like periventricular hemorrhagic infarction (PVHI) (Fig. 1). We propose standardized terminology for classification of fetal intracerebral hemorrhage for use in future research. We also highlight gaps in the available fetal ICH literature to guide future research. Last, we discuss the state of current clinical practice and provide practical guidance for prenatal counseling and postnatal management of fetal intracerebral hemorrhage, recognizing the need for capacity-building in the newly emerging subspecialty of fetal neurology.^5^

## Terminology and classification

Defining specific patterns and subtypes of fetal intracerebral hemorrhage for prenatal application is necessary to advance understanding of pathophysiology, risk factors, and outcomes, including recurrence risk. A lack of consistency in the terminology used in the literature for different types of fetal ICH is a rate-limiting step in the advancement of our understanding of the underlying causes. The terminology used in this review for different patterns of fetal ICH are defined in Table 1.

**Table 1:** Fetal intracranial hemorrhage terminology.

Terminology	Abbreviation	Definition	Timing of diagnosis	Relevant citations
Fetal germinal matrix hemorrhage intraventricular hemorrhage	GMH-IVH	Presence of blood products in the caudothalamic groove or ependyma, and intraventricular blood products diagnosed in a fetus	Antenatal	Dunbar et al. 2020^31^
Fetal hemorrhagic stroke	FHS	Intraparenchymal hemorrhage that appears to be a primary hemorrhage, rather than the secondary hemorrhagic transformation of an ischemic injury	Antenatal	Adapted from Cole et al. 2017^63^
Fetal intraparenchymal Hemorrhage	IPH	Parenchymal hemorrhage identified in a fetus due to any cause	Antenatal	George et al. 2023^43^
Papile grading	N/A	Grading applied to GMH-IVH: Grade I: blood in the ependyma or caudothalamic groove Grade II: blood in the ventricle filling <50% Grade III: blood in the ventricle filling >50% Grade IV: presence of hemorrhage in the periventricular white matter in addition to GMH-IVH (also called periventricular hemorrhagic infarction)	Prior to 34 weeks (either delivered preterm, or fetal)	Papile et al. 1978^64^
Periventricular hemorrhagic infarction	PVHI	Presence of hemorrhage in the periventricular white matter in addition to GMH-IVH, may include evolving encephalomalacia/ porencephaly (also called Papile grade IV)	Diagnosed antenatally or in a delivered preterm infant <34 weeks gestational age	Leijser and de Vries 2019^24^
Periventricular venous infarction	PVI	Cystic encephalomalacia/porencephaly in the periventricular white matter, often with evidence of prior GMH-IVH in the form of hemosiderin staining in the ependymal on susceptibility-weighted imaging	Diagnosed after neonatal period in an infant or child born after 34 weeks gestational age, *presumed* to have occurred prior to 34 weeks.	Kirton and Wei 2010^65^
Porencephaly	N/A	Chronic cystic encephalomalacia of the periventricular white matter causing a “squared” appearance to the ventricle Archaic term often applied to encephalomalacia in other settings; newer term is cystic encephalomalacia	Antenatal or postnatal	Pasternak, Mantovani and Volpe 1980^66^

We highlight GMH-IVH with subsequent PVHI as a distinct entity from IPH to emphasize the different risk factors and pathophysiology (Fig. 1). It is important to acknowledge that in the setting of severe and multiple areas of hemorrhage, it may not be possible to distinguish GMH-IVH with subsequent venous infarction with hemorrhage, and GMH-IVH associated with parenchymal hemorrhage by virtue of the same underlying etiology.

**Fig. 2:**
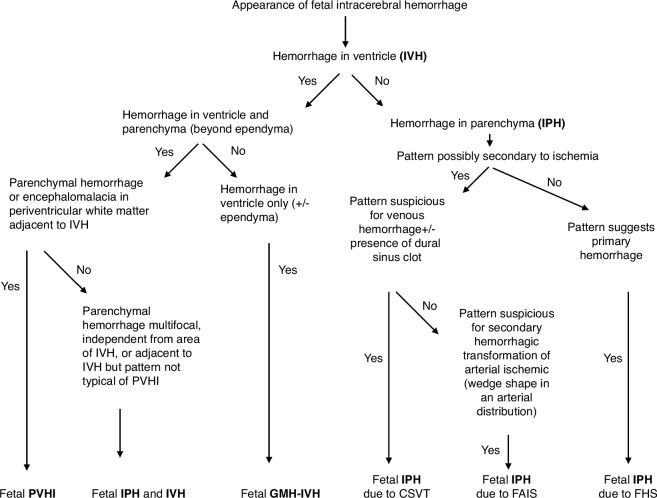
Proposed classification flowchart of fetal intracerebral hemorrhage. CSVT cerebrosinovenous thrombosis, FAIS fetal arterial ischemic stroke, FHS fetal hemorrhagic stroke, GMH-IVH germinal matrix hemorrhage-intraventricular hemorrhage, IPH intraparenchymal hemorrhage, PVHI periventricular hemorrhagic infarction.

In order to provide a standardized fetal ICH classification, we propose a consensus-based approach to classifying fetal PVHI and IPH (Fig. 2). Our first proposed branch point is the presence of intraventricular blood, which may indicate a GMH-IVH, and then whether there is any parenchymal hemorrhage, and whether it is in the classic location for PVHI (periventricular) or distant from the ventricle, which would suggest a separate area of involvement. In the absence of intraventricular blood, we propose three categories based on pattern and etiology if these are possible to determine: primary parenchymal hemorrhage without communication with the lateral ventricle, venous hemorrhagic infarct, or arterial ischemic infarct with hemorrhagic transformation with lesions that do not communicate with the ventricle. Distinguishing these categories may only be possible on postnatal imaging, if ever.

## Epidemiology

The incidence of many fetal neurological conditions is difficult to determine due to the lack of population-based screening after 20 gestational weeks. In many parts of the world, standardized ultrasound anatomy scans are performed at 18–20 gestational weeks, which may be the last time fetal imaging of the pregnancy is performed. Additional ultrasounds may be performed for specific concerns, such as fetal growth, trauma, vaginal bleeding, or a high-risk pregnancy due to multiple gestation, hypertension, diabetes, or other maternal conditions. There are limited epidemiologic data on fetal intracerebral hemorrhage, and studies predominantly report a subset of higher risk pregnancies. Furthermore, different types of fetal intracerebral or even ICH are often combined. Another challenge is that the fetal incidence and birth prevalence may differ given not every fetus with fetal intracerebral hemorrhage will be live born.^3^

Available data estimate an incidence for fetal ICH between 0.1 and 1/1000 pregnancies,^6–8^ while birth prevalence from one 2006 study was estimated to be 0.17–0.35/1000 live births.^9^

## Etiopathogenesis

Diverse causes underlie fetal intracerebral hemorrhage (Table 2) suggesting heterogenous pathophysiology. Recognized causes include congenital infections (i.e., cytomegalovirus, parvovirus B19), autoantibody conditions such as fetal and neonatal autoimmune thrombocytopenia (FNAIT), maternal trauma, cerebral sinovenous thrombosis (CSVT, also known as dural venous sinus thrombosis (DVST)), deep medullary vein thrombosis,^10^ and coagulopathies such as those due to von Willebrand Factor,^11^ congenital factor deficiency (i.e., Factor X), or congenital amegakaryocytic thrombocytopenia due to *MPL* variant.^12–14^ IPH can also be due to genetic conditions that affect blood vessel integrity such as collagen vascular disorders, and the most frequently identified is *COL4A1/2*. Increasingly, a variety of monogenic causes are recognized to underlie a broad spectrum of pediatric cerebrovascular disease and vascular malformations, such as *ACTA2, RASA1*, and *FOXC1*, which can have a perinatal presentation.^15^ In one retrospective single-center study of fetal IPH, genetic disorders were more common (9/44) than congenital infection (2/44) and FNAIT (1/44), and the yield of genetic testing was 39% (9/23).^16^ In contrast, a recent single-center study of fetal cerebellar hypoplasia due to hemorrhage reported acquired etiologies like parvovirus B19 and FNAIT were common, but there were no known cases with genetic etiologies.^17^ Other small series of prenatally detected cerebellar hemorrhage have also reported parvovirus and intrauterine transfusion for fetal anemia as frequently identified etiologies^16,18,19^; however, a cause is not identified in many of the reported cases.^19^ A retrospective multicenter study of FNAIT reported fetal ICH as a complication in 43/592 (7%) pregnancies.^20^

**Table 2:** Possible causes of fetal intracerebral hemorrhage.

Category	Examples
Genetic disorders	*COL4A1* *COL4A2* *COLGALT1* *CTC1* *CTSA* *FOXC1* *HTRA1* *MPL* *NOTCH3* *TREX1*
Infection	Cytomegalovirus (CMV) Parvorvirus Toxoplasmosis Zika Lymphocytic Choriomeningitis Virus (LCMV)
Autoantibody	Fetal and neonatal autoimmune thrombocytopenia (FNAIT)
Vascular malformations	Vein of Galen Malformation Hereditary hemorrhagic telangiectasia PHACE Syndrome (Posterior fossa anomalies, Hemangioma, Arterial anomalies, Cardiac anomalies, and Eye anomalies)
Secondary to venous thrombosis	Fetal cerebral sinovenous thrombosis or fetal dural sinus thrombosis
Secondary to arterial ischemia	
Germinal matrix hemorrhage	Periventricular hemorrhagic infarction (PVHI)
Maternal trauma	Motor vehicle accident
Coagulopathies	Von Willebrand Factor
Pharmacologic/Toxic	Drugs of abuse Warfarin
Idiopathic	

The pathophysiology of fetal GMH-IVH has not been established. In the preterm population, GMH-IVH reflects the sensitivity of the highly vascular and friable germinal matrix to perturbations in blood flow and systemic blood pressure. Most commonly in the lateral ventricles, GMH-IVH is also frequently seen in the cerebellum of preterm infants. After 32–34 weeks the germinal matrix typically involutes, meaning that GMH-IVH is an injury typically diagnosed before 32–34 weeks’ gestation. An important exception to this timeline includes congenital anomalies and genetic syndromes that may delay germinal matrix maturity. For instance, congenital heart disease has been shown to delay germinal matrix maturity by ~2 weeks.^21^ However, the applicability and relevance of the pathophysiologic mechanisms of GMH-IVH in preterm infants to fetuses is not known. Despite this knowledge gap, potential complications of fetal GMH-IVH appear similar to preterm GMH-IVH.^22^ The complications of fetal GMH-IVH include post-hemorrhagic ventricular dilatation (PHVD) and PVHI, a venous infarction of the medullary veins that drain the white matter due to congestion at the level of the germinal matrix.^23,24^ When diagnosed remote from the timing of occurrence with chronic changes on MRI, PVHI is sometimes referred to as periventricular venous infarction. Commonly diagnosed on neuroimaging obtained in infancy or childhood following presentation with early handedness or motor asymmetry, remote PVHI can also be identified on prenatal imaging.^25^ When detected in a child that was born at term, the timing of the GMH-IVH and PVHI is presumed to be antenatal; however, in the absence of serial prenatal and postnatal imaging, determination of the precise timing of a remote venous hemorrhagic infarction cannot be made with certainty. Improved detection of intracerebral hemorrhage prenatally may shed light on this subgroup of children.

### Vascular malformations

Developmental vascular malformations merit specific mention and underscore the importance of accurate diagnosis to inform prenatal counseling and care. Dural sinus malformations (DSMs) are rare vascular malformations in fetuses and infants characterized by venous lakes at the involved dural sinuses, often the torcula, commonly associated with thrombus that can be extensive. Of reported cases of prenatally and postnatally diagnosed torcular DSM (tDSM), parenchymal hemorrhage is present in 8/77 (10%) and other parenchymal injury suggesting prior ischemia in 14/63 (22%).^26^ Natural history data indicate that fetal tDSMs spontaneously involuted in 38/39 cases (97%) with continuation of pregnancy, with 5/38 (13%) initially increasing in size prior to spontaneous involution.^26,27^

Vein of Galen malformations (VGMs) occur when the anterior part of the prosencephalic vein of Markowski does not disappear in its usual developmental course, and instead enlarges due to high pressure of the choroidal feeders, forming a vascular malformation. Diffuse parenchymal injury and hemorrhage have been reported in VGMs.^28^ A recent systematic review of prenatally diagnosed VGMs reports 51 cases from 31 papers, though fetal MRI findings were not reported.^29^

### Risk factors

Risk factors for fetal intracerebral hemorrhage are challenging to discern because of ascertainment bias due to the indication for fetal imaging. As above, a variety of potential fetal and maternal concerns may prompt additional imaging beyond the 20-week anatomy scan, and therefore may appear to be associated with fetal ICH without a causal link.

Male sex has been identified as a risk factor for fetal hemorrhage to varying degrees. In a systematic review of fetal GMH-IVH, the sex proportion among those with PVHI was equal.^22^ The proportions of males in several articles of fetal ICH (including extracerebral hemorrhage), have ranged from 57 to 77%.^2,16,30^ In delivered preterm infants, a preponderance of males with GMH-IVH and those with PVHI is acknowledged.^23,24^ Similarly, in term-born children diagnosed with  remote PVHI, males are over-represented.^31^ Sex effects are also reported in animal models of perinatal hemorrhage and stroke,^32^ underscoring the importance of understanding how sex effects may influence the risk of hemorrhage, as well as outcomes.

Possible risk factors for fetal PVHI were evaluated in a systematic review of 80 studies comprising 105 fetuses with PVHI, including twin pregnancy, congenital anomalies, small for gestational age and/or intrauterine growth restriction. In over one-third there were no associated abnormalities.^22^ Twin-twin transfusion syndrome and intrauterine transfusion are common risk factors for fetal IPH.^16^

## Neuroimaging

### Prenatal sonography

The most common sonographic finding when fetal intracerebral hemorrhage is present is ventriculomegaly. The blood clot in GMH appears as an echogenic collection similar to the normal choroid plexus, and may be adherent to choroid plexus or separate.^33^ It can be challenging to estimate the amount of intraventricular blood present due to fetal motion, though more severe GMH-IVH tends to be associated with enlargement of the lateral ventricles over time. Progressive development of complex texture is typical over subsequent days, such as hyperechoic nodular ependyma or irregular, or heterogeneous echogenicity of the choroid plexus.^33^ Small GMH typically resolve spontaneously over time. Depending on the time interval between PVHI or IPH occurrence and ultrasound detection, there may be findings of increased echogenicity in the periventricular white matter or parenchyma, or evidence of cystic evolution and porencephaly.^33,34^ Fetal cerebellar hemorrhage in particular may be underestimated due to technical challenges in assessing the posterior fossa prenatally. Often when detected by ultrasound, fetal cerebellar hemorrhage is unilateral and associated with cerebellar hypoplasia. Neurosonography can provide greater resolution when a suspected intracranial abnormality is detected on routine sonography.^34^

Many studies have applied the Papile grading scheme from the preterm population to fetal GMH-IVH^2,22^; however, this approach is limited by the potential time interval between the occurrence of fetal GMH-IVH and when it is detected on prenatal imaging, which may be longer compared to preterm infants. In turn, this time interval likely impacts assessment of the amount of hemorrhage present in fetal GMH-IVH, as well as the ability to distinguish acute ventriculomegaly related to hemorrhage from fetal post-hemorrhagic ventricular dilatation. A recent paper proposed description of location of IVH, presence of parenchymal hemorrhage, ventriculomegaly and other associated findings rather than a grading system for fetal GMH-IVH.^1^

Systemic findings on ultrasound may also provide additional phenotypic clues pointing toward a specific syndrome or genetic etiology. For example, the presence of arthrogryposis may suggest a collagen vascular disorder, whereas organomegaly, hydrops and peritoneal calcifications can be seen in congenital infection.

### Fetal MRI

The addition of fetal MRI has been found to improve diagnostic accuracy of suspected abnormalities on prenatal ultrasound;^35,36^ however, the diagnostic accuracy of fetal MRI for ICH has not been systematically evaluated. Recent studies have shown that identification of ICH on fetal MRI is often preceded by a different referral diagnosis based on ultrasound, most commonly ventriculomegaly. Only 6/57 (11%) cases of fetal ICH confirmed by fetal MRI in one study were preceded by a referral diagnosis of ICH based on ultrasound,^3^ whereas 12/22 (54%) were suspected to have fetal ICH based on ultrasound in a separate single-center study.^2^ In a series of 306 fetuses where ventriculomegaly was the only abnormality on ultrasound, five (2%) were found to have ICH on fetal MRI, which was confirmed postnatally.^37^ Other studies highlight the complementary nature of ultrasound and MRI for fetal intracerebral hemorrhage. Targeted neurosonography has shown superior sensitivity for detecting periventricular changes in the acute phase of PVHI, IVH, and basal ganglia or thalamic involvement compared to MRI in certain cases.^34^ Ultrasound is also reported as more reliable for determining the timing of IVH in some retrospective case series.^38,39^

**Fig. 3:**
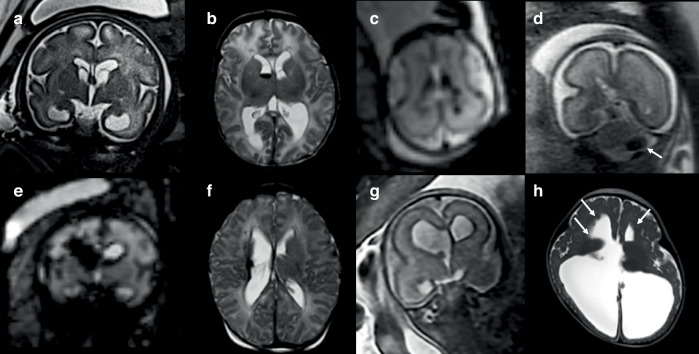
Intracerebral hemorrhage on fetal MRI. Coronal T2-weighted MRI (**a**) at 33 gestational weeks showing mild ventriculomegaly with cysts at the bilateral caudothalamic grooves, and postnatal axial T2-weighted MRI in this case (**b**) showing small germinal matrix hemorrhage, septation in the right lateral ventricle and white matter hyperintensities due to congenital CMV. Axial echoplanar imaging (EPI) at 22 gestational weeks (**c**) in monochorionic twin showing bilateral germinal matrix-intraventricular hemorrhage (GMH-IVH), with cerebellar hemorrhage (**d**) on coronal T2-weighted MRI. GMH-IVH with right periventricular venous hemorrhagic infarction (**e**) at 31 gestational weeks due to *COL4A2* variant, and postnatal axial T2-weighted MRI showing chronic changes (**f**). Twin with ventriculomegaly on coronal T2-weighted MRI (**g**) at 22 weeks and bilateral GMH confirmed on EPI (not shown), with postnatal follow-up MRI at 8 months (**h**) showing persistent severe ventriculomegaly despite ventriculoperitoneal shunting for post-hemorrhagic hydrocephalus with parenchymal volume loss and periventricular nodular heterotopia.

Findings on fetal brain MRI that indicate acute or subacute hemorrhage include the presence of T1 hyperintense and T2 hypointense lesions, which may distort surrounding tissue due to local mass effect.^16,33^ The approximate timing of hemorrhage by T1- and T2-shortening combinations is difficult due to the high concentration of fetal hemoglobin. Chronic changes associated with hemorrhage include porencephaly, which may have areas of signal change suggestive of prior hemorrhage. An additional challenge posed by fetal MRI is the quality of hemosiderin specific sequences, as gradient echo (GRE) and susceptibility weighted imaging are particularly susceptible to motion artifact. Echo planar imaging (EPI) in fetal MRI is typically used for faster acquisition times, which may decrease sensitivity for hemorrhage.^40–42^ Examples of fetal ICH on fetal MRI and follow-up imaging are shown in Fig. 3.

In one single-center retrospective study (*n* = 44) of fetal IPH, fetal MRI showed predominantly supratentorial and focal hemorrhage, with diffuse non-hemorrhagic injury in 43%.^16^ The frontal lobes (80%) and/or the parietal lobes (52%), and/or the deep gray nuclei (45%) were most commonly involved. IPH was commonly associated with GMH-IVH and ventriculomegaly; however, the authors reported that it was not possible to reliably grade severity of GMH-IVH or classify PVHI.^16^ Cortical malformations associated with IPH were seen in one-third of cases. Repeat fetal MRI after a median of 3 weeks showed expected evolution of hemorrhage in the majority (7/8), and one case showed a new finding of polymicrogyria overlying the IPH. The ratio of the maximal extent of hemorrhage to biparietal diameter was proposed to compare severity of hemorrhage on fetal MRI across different gestational ages.

The intrinsic characteristics of the hemorrhage can be helpful in determining etiology and guiding investigations. For instance, hemorrhage due to *COL4A1/2* mutations classically results in a combination of IVH and frontal lobe parenchymal hemorrhage, often of different ages and resulting in porencephaly, which can be unilateral or bilateral.^16,43^ Multifocal white matter injury can also be seen and tends to accumulate over time. In FNAIT, fetal GMH-IVH, PVHI, IPH, and multi-compartment hemorrhage have been reported, with more than half (23/43) diagnosed <28 gestational weeks and multi-phasic hemorrhage occurring in 12% (5/43).^20^

### Utility of postnatal imaging

Postnatal neuroimaging with MRI provides higher resolution images as well as hemosiderin specific sequences to fully visualize areas of parenchymal hemorrhage. Additionally, MRI allows for better visualization of the full-term cortical development; as many fetal MRI studies are performed mid-gestation, there may be malformations of cortical development either associated with or as a disruptive consequence of parenchymal hemorrhage, which will affect prognosis and investigations. New findings on neonatal MRI were frequent in cases of severe fetal IPH with or without IVH that underwent postnatal brain MRI in one study (6/13).^16^ These findings support the role of postnatal MRI to detect additional complications related to fetal IPH, such as recurrent hemorrhage, progression of ventriculomegaly, polymicrogyria, or additional white matter injury unrelated to fetal IPH.^16^ There are no systematic data on routine postnatal MRI after fetal intracerebral hemorrhage, and such data are requisite to quantify the probability of new clinically relevant findings. Although these data are limited currently, postnatal MRI is not thought to be needed after fetal low-grade GMH-IVH in neonates and infants that are otherwise clinically well. Postnatal vascular imaging (MRA, MRV, angiogram) may also be helpful diagnostically in cases of vascular malformation, and for endovascular intervention.

## Outcomes

There is limited literature on outcomes after fetal ICH. This body of literature is retrospective and prone to selection bias. The generalizability of the rates of different pregnancy, perinatal, and neurological outcomes should be interpreted with caution. There are two systematic reviews and meta-analyses that report outcomes of fetal ICH, with most studies based on prenatal ultrasound. Sileo et al. report perinatal and neurodevelopmental outcomes in 193 fetuses from 16 studies inclusive of any type of ICH on prenatal imaging.^44^ Of these 16 studies, 12 overlap with the meta-analysis by Dunbar et al.^22^ that reports 240 cases of GMH-IVH and PVHI from 80 articles, including case reports and case series. Of the 4 studies included by Sileo et al.^44^ without overlap by Dunbar et al.,^22^ there is a retrospective series of FNAIT,^20^ 2 small series on fetal cerebellar hemorrhage,^18,19^ and a series on fetal subdural hemorrhage.^45^ Whereas the meta-analysis by Sileo et al.^44^ combines Grade III GMH-IVH with PVHI (referred to as Grade IV), the Dunbar^22^ meta-analysis distinguishes between GMH-IVH with or without parenchymal hemorrhage. A few additional studies have been published since the publication of both meta-analyses. Here, we aim to integrate a discussion of this recent literature with the outcomes reported by meta-analysis.

### Pregnancy and perinatal outcomes

Data on pregnancy outcomes report variable rates of termination of pregnancy (TOP), and do not describe reasons for termination when intracerebral hemorrhage is detected. Broadly, rates of TOP are higher when parenchymal hemorrhage is present. TOP is reported in 13/104 (13%) cases of GMH-IVH without parenchymal injury and 23/105 (22%) to 17/33 (52%) for PVHI.^22^ In one study of fetal IPH, TOP occurred in 16/44 (36%) of cases, and was strongly associated with maximal diameter of IPH.^16^ Meta-analysis data report TOP in 4/24 (17%) fetuses with infratentorial hemorrhage, and a more recent single-center study reported TOP in 2/16 (15%).^17,44^ Spontaneous intrauterine demise is low overall, but higher than population-level rates, occurring in 6/104 (6%) of cases of GMH-IVH without parenchymal injury, 9/105 (9%) with PVHI, 5/58 (9%) with IPH, and 2/24 (8%) with infratentorial hemorrhage.^16,22,44^

Preterm birth is common, both spontaneous and medically induced, occurring in 39/75 (52%) with GMH-IVH, 21/69 (30%) with PVHI,^22^ 5/21 (24%) with IPH,^16^ and 2/16 (13%) with cerebellar hemorrhage.^17^ Medically induced indications for preterm delivery may include worsening severity of ventriculomegaly, and progressive ischemia or hemorrhage due to venous sinus thrombosis of vascular malformations. Whether preterm birth is spontaneous or medically induced is not typically reported, although would be informative to include in future studies. Postnatal mortality is often related to prematurity, systemic malformations, or other comorbidities, reported to occur in 8/104 (8%) with GMH-IVH, 7/105 (7%) with PVHI,^22^ and 2/21 (10%) with IPH.^16^ Antenatal counseling should highlight the risk of prematurity as a potentially important modifier of the risk of adverse neurodevelopmental outcomes.

Available data on the relationship between etiology of intracerebral hemorrhage and pregnancy outcome are limited to FNAIT. In a retrospective study of FNAIT,^20^ intrauterine demise occurred in 5/43 (12%) pregnancies complicated by fetal ICH. Preterm delivery occurred in 29/38 (76%), and perinatal or postnatal death occurred in 10/38 (26%) cases of fetal ICH due to FNAIT.

### Neurological outcomes

Selected studies of neurological outcomes after fetal intracerebral hemorrhage are summarized in Table 3. The most common complication in surviving infants is motor impairment, seen in 22/80 (28%) of those without clear evidence of parenchymal hemorrhage, and 35/47 (75%) with PVHI and 12/13 (92%) with IPH.^16,22^ Many studies on isolated GMH-IVH have reported mild motor impairments (e.g., transient tone abnormalities, dyspraxia), whereas more severe impairment (e.g., cerebral palsy) may occur in parenchymal hemorrhage. The location and extent of parenchymal hemorrhage tends to correlate with the topology of motor impairment^16^; however, the relationship between fetal MRI findings, timing of hemorrhage, and the degree of functional motor impairment is not known. Cerebral palsy was reported in 2/16 (13%) cases of cerebellar hemorrhage in the meta-analysis by Sileo et al.,^44^ and there were no cases of motor impairment in a more recent single-center cohort.^17^

**Table 3:** Selected studies on fetal intracranial hemorrhage.

Study focus	Study	Design	Population	*N*	Outcome/measurement	Duration of Follow up	Key Findings
Role of MRI	Sanapo et al. 2017^30^	Retrospective single center cohort	Fetal ICH 2008-2015	36	Pregnancy outcome	N/A	- IUFD: 1/24 (4%) GMH; 7/12 (58%) non-GMH - TOP: 5/24 (21%) GMH; 1/12 non-GMH - Live: 18/24 (75%) GMH; 4/12 (33%) non-GMH
Patterns, etiology, outcomes	Vassar et al. 2024^16^	Retrospective single center cohort	Fetal IPH 1996-2022	44	Cerebral palsy, developmental delay, epilepsy	Median 7 years	- Etiology: TTTS (*n* = 10), COL4A1/2 (*n* = 8), other maternal/fetal (*n* = 10), intrauterine transfusion (*n* = 5), unknown (*n* = 11) - Pregnancy outcome: TOP 16/43 (37%), IUFD 3/43 (7%), radioablation 2/43 (5%), livebirth 22/44 (50%), premature 5/22 (23%), unknown 1/44 (2%). - Neurologic outcomes: CP 12/13 (92%), GDD/ID 6/13 (46%), Epilepsy 5/13 (38%)
Neurodevelopmental outcomes	Eldad et al. 2024^2^	Retrospective single center cohort	Fetal ICH 2011-2016	22	Vineland-II Adaptive Behaviour Scales (VABS)	Median 19 months	- Imaging findings: IVH 91%, infratentorial 4.5%, IVH and infratentorial 4.5% - Pregnancy outcome: TOP 50%, liveborn 50% - VABS: normal 9/11 (82%), moderately low 2/11 (18%)
Neurodevelopmental outcomes	Scelsa et al. 2022^17^	Retrospective single center cohort	Fetal cerebellar hypoplasia due to hemorrhage 2010-2020	16	GRIFFITHS II, WPPSI-III, or WISC-IV according to the child’s age	Median 3.5 years	- Median age at diagnosis 28.5 weeks (range 21–34 weeks) - Pregnancy outcome: termination 2/16 (12.5%), liveborn 14/16 (87.5%), preterm 2/16 (12.5%) - Etiology: parvovirus B19 2/14 (14%), FNAIT 2/14 (14%), transfusion 3/14 (21%), multiple risk factors 4/14 (29%) - Neurodevelopment: normal 6/10 (60%), mild dyspraxia and visuoperceptual deficit 3/10 (30%), autism spectrum 1/10 (10%)
Comorbid conditions, mortality, Neurodevelopmental outcomes	Dunbar et al. 2021^22^	Systematic review and individual patient meta-analysis	Fetal GMH-IVH 1982-2018	240	Pregnancy outcome, shunt placement, motor impairment, developmental delay, epilepsy	12 months	- Median age at diagnosis 31 weeks - Pregnancy outcome: liveborn 179/240 (75%), preterm 60/127 (47%), IUFD 16/231 (7%), TOP 36/231 (16%). - Prenatal imaging findings: Grade I/II GMH-IVH 11.6%, grade III 43.1%, PVHI 45.3%. - Postnatal death: 22/240 (9%) - VP shunt: 66/139 (47%) - Motor impairment: 57/127 (45%) - Epilepsy: 17/130 (13%)
Fetal CST measurement and neurodevelopment outcomes	Hadi et al. 2024^1^	Retrospective single center cohort	Fetal GMH-IVH 2011-2018	35	Morphology and integrity of CST and the relationship with neurodevelopment outcomes	Mean 7.2 years	- 19/35 (54%) with parenchymal injury - CST involvement correlated with parenchymal injury - Motor impairment in 1/7 (14%) with intact CST and 5/5 (100%) with impaired CST
Neurodevelopmental outcomes	Kim et al. 2024^67^	Retrospective single center cohort	Fetal GMH-IVH 2012-2023	27	VP shunt placement, motor impairment, neurologic impairment, developmental delay, epilepsy	Mean 40.1 months	- VP shunt in 9/16 (56%) - Motor impairment 13/16 (81%) - Neurologic impairment 6/16 (38%) - Developmental delay 9/16 (56%) - Epilepsy in 11/16 (69%)
Mortality and neurodevelopment outcomes	Sileo et al. 2022^44^	Systematic review and individual patient meta-analysis	Fetal ICH 2000-2020	161	Mortality, need for shunt, CP, neurodevelopmental outcomes		- Imaging findings: IVH 48%, IPH 22% (12 supratentorial, 24 infratentorial), complex 11% - Perinatal death in 26/161 (16%) - Shunt in 14/44 (32%) with IVH, 2/18 (11%) for IPH - CP in 25/97 (26%) intra-axial (32% with IVH, 25% with IPH) - Mild NDD in 19/97 (20%) intra-axial (5/44 IVH, 2/20 for IPH) - Severe NDD in 33/97 (34%)
Mortality and neurodevelopment outcomes	Gupta et al. 2022^3^	Retrospective single center cohort	Fetal ICH 2012-2020	50	Only 10% of referrals were for suspected ICH	Mean 1.8 years	- Development normal in 21/37 (56%) - Epilepsy in 4/37 (11%)
Genetic causes	Lecca et al. 2023^68^	Regional case series	Prenatal *ESAM* gene mutation	8	Mortality, NDD, CP, epilepsy		- Prenatal imaging: VM (*n* = 2), ICH (*n* = 2), PVHI (*n* = 3), porencephaly (*n* = 1) - Pregnancy outcome: in utero demise (*n* = 1), TOP (*n* = 3), live birth (*N* = 4) - Neurological outcome: epilepsy 2/4 (50%), CP 2/4 (50%), GDD 2/4 (50%)
Genetic causes	George et al. 2023^43^	Retrospective single center cohort	Fetal IPH with *COL4A1/2* 1998-2022	8	Imaging findings, motor outcomes	At least 12 months	- Fetal MRI: small/unifocal to multifocal and bilateral IPH in the frontal lobes and basal ganglia; hemorrhagic porencephaly 4/8 (50%) - Pregnancy outcome: TOP 3/8 (37.5%), liveborn 5/8 (62.5%), preterm 1/5 (20%) - All with clinical follow up had CP
Genetic causes	Ilves et al. 2023^69^	Retrospective single center cohort	Fetal PVHI 1994-2019	6	Genetic diagnosis, motor and epilepsy outcomes		- 2/6 had collagenopathy - Collagenopathy associated with bilateral multifocal stroke, greater motor impairment and epilepsy
Fetal and postnatal torcular dural sinus malformations (tDSM)	Yang et al. 2018^26^	Review and meta-analysis	Prenatal and postnatal tDSMs	77 prenatal and 22 postnatal	Imaging findings, clinical outcomes		- 36% of prenatal and 96% of postnatal tDSMs had evidence of arterialization - DSMs spontaneously involuted in 38/39 (97%) cases where pregnancy continued - TOP 17/77 (22%) prenatally diagnosed - Favorable prognostic factors: absence of evidence for arterialization, decrease in tDSM size, presence of clot, documented increase in clot size/percentage, absence of ventriculomegaly, and absence of parenchymal injury. - Vascular intervention in 14/58 (24%) prenatal diagnoses born live versus 16/22 (73%) with postnatal diagnosis
Fetal vein of Galen malformations (VGMs)	Di Meglio et al. 2024^29^	Systematic review	Prenatally diagnosed VGMs	51	Imaging findings, clinical outcomes		- Common prenatal features were fetal hydrocephalus 20/51 (39%) and cardiomegaly 29/51 (56%), with fetal heart failure in 19/29 (66%) - Associated anomalies 6/51 (12%): poly- hydramnios (*n* = 2), oligohydramnios (*n* = 1), VACTERL (*n* = 1), recurrent hydrothorax (*n* = 1), adrenal hemorrhage (*n* = 1) - Pregnancy outcome: TOP 4/51 (8%), live birth 43/51(84%), unknown 4/51 (8%) - Neonatal outcome: 33/42 (79%) admitted to NICU on day 1 with heart failure in 29/33 (89%), respiratory distress in 2/33 (6%), sepsis 1/33 (3%), hydrocephalus 1/33 (3%). Ten infants (21%) were discharged with plan for elective embolization. - Mortality: Overall mortality was 25/43 (48%). Of those with heart failure, 20/29 (69%) died before embolization could be performed. - Morbidity: Of the survivors, 14/18 (78%) reported to have normal development, mild disability in 2/18 (11%) and severe disability in 2/18 (11%)

*CST* corticospinal tract, *CP* cerebral palsy, *GDD* global developmental delay, *GMH* germinal matrix hemorrhage, *IUFD* in utero fetal demise, *ICH* intracranial hemorrhage, *IPH* intraparenchymal hemorrhage, *IVH* intraventricular hemorrhage, *NDD* neurodevelopmental disorder, *PVHI* periventricular hemorrhagic infarction, *tDSM* torcular dural sinus malformation, *TOP* termination of pregnancy, *VGM* vein of Galen malformation, *VM* ventriculomegaly, *VP* ventriculoperitoneal.

Little is known about cognitive and language outcomes after fetal intracerebral hemorrhage. Several factors likely contribute to variability in outcomes, including location of hemorrhage and its correlation with functional areas, involvement of critical brain areas (e.g., cerebellum, basal ganglia), and severity of ventriculomegaly. Underlying etiology additionally contributes to outcome in fetal IPH and fetal IVH, likely mediated in part by risk of recurrent hemorrhage and risk of prematurity, as well as medical complexity. However, the currently available literature has not specifically examined the influence of these factors on neurodevelopmental outcomes. Meta-analysis data indicate that developmental delay was present in 13/75 (21%) children with history of fetal grade III GMH-IVH, and 28/77 (57%) children with fetal PVHI, whereas none of the cases with grade I/II GMH-IVH had developmental delay.^22^ A single-center study reported intellectual disability or global developmental delay in 5/13 (38%) with IPH on fetal MRI.^16^ Neurodevelopmental delay was reported in 5/16 (31%) cases of cerebellar hemorrhage in the meta-analysis by Sileo et al.^44^ One small single center study of 10 patients evaluating outcome at a median 3.5 years (range 1–8) after isolated cerebellar hemorrhage found 6 had normal neurodevelopment and cognition, and three had mild dyspraxia and visuoperceptual impairment and one had autism spectrum disorder.^17^

Few studies have described epilepsy outcomes in fetal intracerebral hemorrhage. Epilepsy is reported in 3/74 (4%) with GMH-IVH, 13/50 (26%) with PVHI in a systematic review with a median follow up of 12 months (range 0–96),^22^ and 5/13 (38%) with IPH with a median follow up of 7 years.^16^

### CSF diversion

Requirement for CSF shunting is reported in 31/77 (40%) of cases with fetal GMH-IVH without parenchymal injury, 34/57 (60%) with PVHI, and 2/21 (10%) with IPH.^16,22^ Progression of ventriculomegaly in utero is associated with shunt placement postnatally.^22^ The rates of shunting after fetal GMH-IVH and PVHI appear higher than the preterm population. It is uncertain to what extent ascertainment bias in the reported literature contributes to this difference, as cases with progressive or more severe fetal ventriculomegaly may be more likely to be identified. Another potential reason for the differential rates of CSF diversion in fetuses compared to the preterm population is the longer duration of progressive ventriculomegaly in utero before intervention is possible postnatally.

### Recurrence risk

Fetal IPH and other ICH patterns may be associated with ongoing recurrence risk perinatally and postnatally if there is an underlying condition such as *COL4A1*, other collagen vascular disorders, and those with vascular anomalies.^43,46^ In contrast, the recurrence risk is near zero in perinatal arterial ischemic and hemorrhagic stroke.^47^

## Current practice and practical guidance for pediatric neurologists

We surveyed pediatric neurologists that provide fetal consultations across the United States in 2023.^5^ Among 43 individual institutional responses, there was substantial variability in the landscape of current practice. The majority of respondents reported providing fetal neurologic consultation in collaborative care with a prenatal diagnosis center. Fewer than half of respondents (40%) reported having relevant subspecialty training, and annual consultation rates varied markedly across centers, indicating different levels of experience in centers with access to prenatal neurology consultation. Nearly all respondents indicated an interest in educational initiatives and ranked clinical practice guidelines as their top priority for this developing field. Recognizing the growing demand for fetal neurological care,^5^ and limited access,^48^ we outline practical considerations for management and counseling of fetal intracerebral hemorrhage to facilitate capacity-building, standardization of care, and collaborative research. These practical recommendations represent the current practice and consensus expert opinion of this international working group of pediatric neurologists that provide fetal neurological consultation and postnatal follow-up care.

### Antenatal perinatal management

#### Fetal imaging

Fetal MRI is preferred following detection of suspected hemorrhage on fetal ultrasound. Imaging should be performed in a center with protocols optimized for fetal neuroimaging and interpreted by a neuroradiologist with expertise in fetal neuroimaging. Hemosiderin detection sequences should be included. After the diagnosis of intracerebral hemorrhage, longitudinal surveillance with serial fetal ultrasound is needed to monitor ventriculomegaly, and fetal head circumference, as well as to assess for new hemorrhage. Detailed anatomy scan is recommended to assess the extent of fetal abnormalities and determine whether a specific syndrome may be present.

Repeat fetal MRI may be informative in select cases, including when the initial MRI is obtained early in gestation and there are potential pregnancy management decisions, as well as to evaluate new parenchymal findings on fetal ultrasound, or to examine the extent of parenchymal hemorrhage and the secondary developmental consequences.

#### Family history

Elements of the family history that may support *COL4A1/2*-related disorders include ICH, porencephaly, unilateral cerebral palsy, focal epilepsy, renal disease, cataracts, and early-onset vascular dementia or stroke. It should be noted that over half of cases are de novo and due to variable penetrance and expressivity, family history is often unrevealing. A family history of bleeding disorders, vascular malformations, cutaneous vascular lesions, and stroke in the young should also be elicited.

#### Etiologic testing

There is no evidence base to recommend specific investigations; however, expert opinion recommends serological testing for infections such as CMV, parvovirus when cerebellar hemorrhage is present, other infectious etiologies as indicated case-by-case (i.e., Toxoplasma, LCMV and Zika virus), as well as parental antibody testing for FNAIT. Amniocentesis should be offered in all cases to enable testing for infection and genetic testing.

When evidence of parenchymal hemorrhage is present, genetic testing is recommended. If available, broad testing with exome or genome sequencing may be informative,^11^ and should be considered in all cases. If broad sequencing is not accessible and feasible, consider obtaining *COL4A1*/*2* single gene testing or a cerebral small-vessel disease panel. Expectant parents should be counseled about the low pretest probability of genetic testing in the setting of isolated low-grade GMH-IVH without parenchymal hemorrhage. Practice patterns in some centers include offering broad sequencing to any pregnancy with an intracranial abnormality; however, access varies widely.

#### Fetal neurology consultations

Consultations should be conducted in collaboration with maternal-fetal medicine, to present the available diagnostic and prognostic information, as well as acknowledge uncertainties in etiology and prognosis. Studies of parents faced with a fetal diagnosis voice that having comprehensive information, delivered in plain language by a compassionate professional as key elements to reduce their stress.^49–53^ Parents should be explicitly reassured that they are not responsible for the ICH, which is often an unvoiced fear. Pediatric neurosurgery consultation should be obtained in cases of progressive ventriculomegaly where postnatal intervention may be needed.

#### Psychosocial support for expectant parents

Early support from a social worker and counselors/perinatal psychologists is advised to provide psychosocial support to expectant parents through the process of investigations. Perinatal palliative care and neonatology consultations should be considered in cases with anticipated lifelong consequences, and cases with uncertain consequences to facilitate discussions regarding perinatal planning, prognostic uncertainty, and family support. Referral to grief counseling is recommended as available for fetal demise and TOP.

#### Timing, location, mode of delivery

Cesarean section is recommended in FNAIT.^54^ There is otherwise no evidence available to guide mode of delivery and when to deliver the infant, however head size and risk of extension of hemorrhage should be considered in multidisciplinary discussion, and some centers routinely recommend cesarean section for fetal ICH independent of etiology. There is insufficient evidence to recommend scheduling early delivery in the case of progressive ventriculomegaly, and practice varies widely.^55^ Important considerations in planning the location of delivery include the need for specialized services like neurosurgery and tertiary level care. The indications for tertiary care delivery should be explained to expectant parents. For small, non-progressive hemorrhages with an otherwise healthy fetus, delivery at a specialized center is not required. If intensive care is anticipated post-delivery, it is recommended that the parent(s) meet with a neonatologist.

### Postnatal recommendations

#### Neonatal evaluation

General examination should be performed with attention to the head circumference, fontanel, and sutures, as well as assessment for dysmorphisms, ocular findings, arthrogryposis, cutaneous vascular findings, and stigmata of congenital infection. A complete neurological examination should be performed. If encephalopathy is present or there are suspected clinical seizures, continuous electroencephalography (EEG) should be obtained as available.^56^ These signs could reflect new acute injury (i.e., hemorrhage, hypoxia-ischemia, ischemia) related or unrelated to the prenatal presentation, new postnatal infection, or an underlying genetic or metabolic disorder. Clinical signs such as a bulging fontanel or rapidly increasing head circumference should prompt neuroimaging (see below) and neurosurgical referral.

#### Neuroimaging

All infants with history of fetal GMH-IVH and/or IPH should have a postnatal cranial ultrasound, ideally prior to discharge from the birth hospitalization to establish a postnatal baseline and inform the need for ongoing monitoring of the ventricles. Surveillance of ventriculomegaly with serial ultrasound should be individualized in discussion with pediatric neurosurgery based on risk for progression to hydrocephalus. Postnatal MRI (3T if available) should be obtained in cases with parenchymal hemorrhage, whether confirmed on fetal imaging or suspected on postnatal cranial ultrasound. MRI should be performed within the first 3 months of age using the “feed and bundle” method, as feasible, to avoid need for anesthesia. Magnetic resonance angiography (MRA) and magnetic resonance venography (MRV) should be considered on a per-case basis. Resolution of fetal low-grade GMH-IVH on postnatal cranial ultrasound does not necessitate further imaging. In our experience some families may find follow-up MRI to confirm the absence of new hemorrhage or additional findings to be of substantial reassurance.

#### Other testing

The timing and type of investigations after delivery can be adjusted based on the timing of delivery, the condition of the infant, including associated anomalies, and the progression of findings antenatally. Genetic testing should be obtained in cases of parenchymal hemorrhage when there is not an otherwise apparent cause, such as multiple gestation or congenital infection, and genetic testing was not performed prenatally. Selection of exome or genome sequencing versus a cerebral small vessel gene panel may be based on regional access. CMV testing should be performed within 3 weeks of delivery if no confirmed etiology is otherwise established, and CMV was not definitively excluded with amniocentesis. Parvovirus testing should be performed in cases of prenatal cerebellar hemorrhage if not excluded prenatally.

Basic baseline coagulation studies, including platelets, INR, PT, PTT, should be obtained when parenchymal hemorrhage is present. Consultation with hematology can guide workup for a specific hematologic cause of hemorrhage, as well as guide the timing of specific tests that may be more reliable after the neonatal period. Further parental testing may be indicated in cases of FNAIT to prevent recurrence.

If findings are suggestive of collagen vascular disorder, including bilateral porencephaly, mixed hemorrhagic and ischemic findings, and hemorrhages of different ages,^43,57^ or there is a suggestive family history, then a comprehensive multi-system examination is warranted, including ophthalmological exam, renal ultrasound, creatine kinase (CK), and electrocardiogram in addition to confirmatory genetic testing.

#### Neurodevelopmental follow-up

Pediatric neurology follow-up is needed to monitor neurodevelopment, and for the occurrence of seizures. Close surveillance of head circumference is needed in children with risk of progressive ventriculomegaly after fetal ICH, and collaborative care with pediatric neurosurgery is recommended. A suggested schedule for neurological assessment could mirror other at-risk populations with follow-up at 3 months, 6 months, 12 months, 24 months, preschool age, and then at school age, with more frequent visits as needed when there are neurodevelopmental impairments or other neurological diagnoses requiring management such as epilepsy. Early intervention services with physical, occupational, speech and/or vision therapy should be implemented as needed to support neurodevelopment.

## Practice and research gaps

### Terminology

Formalized consensus across disciplines around terminology used to describe fetal intracerebral hemorrhage and associated imaging findings like cystic evolution is needed. Building consensus among pediatric neuroradiologists with expertise in fetal MRI, maternal-fetal medicine, and pediatric neurologists using a robust method like the Delphi technique,^58,59^ is a foundational step to address practice and research gaps.

### Epidemiology

The incidence of fetal GMH-IVH with or without subsequent PVHI and fetal IPH are not well known. The reasons for the apparent male predominance are not known. Even less data is available on the incidence of less common pathologies, such as DSM.

### Fetal imaging

Improved sensitivity of fetal MRI for blood products may improve diagnosis of fetal IPH. This may be of particular use for the detection of hemorrhage in cases of secondary disruptive malformations of cortical development such as schizencephaly and polymicrogyria. Use of consensus terminology will facilitate collaborative multicenter studies to describe the pattern and location of hemorrhage(s) on MRI, and associated findings, as well as how these factors relate to different etiologies and outcomes. Such datasets could be leveraged to develop and evaluate fetal GMH-IVH and IPH grading systems and compare the predictive value for outcomes with the Papile grading system in this population.^1^ The predictive value of imaging indices of preterm PHVD requiring neurosurgical intervention should also be evaluated in the fetal population.

### Etiopathogenesis

The yield of genetic testing with targeted vasculopathy panels and whole exome or genome sequencing is not known. The yield of testing for non-genetic etiologies including congenital infections and FNAIT is not established and may vary regionally. Although prior studies have suggested a low likelihood of an identifiable etiology in low-grade fetal GMH-IVH, many of these studies are not contemporary, and it is possible that underlying genetic and non-genetic etiologies are underrecognized in this subgroup. Integrating placental pathology in studies of etiology may also provide important insights.

### Mode and timing of delivery

The risk of extension in fetal ICH through a trial of labor and vaginal delivery is not known. However, a Cesarean section is routinely recommended in FNAIT because of suspected high risk of extension in this condition.^54^ How timing of delivery in cases with severe ventriculomegaly due to PHVD and timing to cerebrospinal fluid diversion may relate to outcome is not known. Mounting data in the preterm population show that earlier intervention for PHVD is associated with improved neurodevelopmental outcomes.^60–62^ Future studies examining the relationship between the duration of PHVD and outcome in the fetal population are needed. The influence of other clinical factors on clinical decision-making regarding medically-induced preterm delivery are not known.

### Postnatal imaging

The yield of postnatal MRI is not established. For selected cases, such as arteriovenous malformation, or DSM, a postnatal neurovascular study may be needed to determine the etiology or neurovascular or surgical interventions. Neonatal MR angiogram/venogram (MRA/MRV) using a time-of-flight sequence may have low resolution without contrast, though the risk/benefit ratio of contrast in the neonatal period is not established.

### Outcomes

Available literature on neurodevelopmental outcomes is limited. Prospective multicenter studies are needed to understand developmental and cognitive outcomes in childhood and adolescence, as well as risk of epilepsy, and rates of CSF diversion. How the location of hemorrhage and its correlation with functional areas, involvement of critical brain areas (e.g., cerebellum, basal ganglia), and severity of ventriculomegaly, relate to outcome will require larger sample populations to better understand. Given the prevalence of prematurity after fetal ICH, the influence of gestational age on clinical and neurodevelopmental outcomes of fetal ICH outcomes should be further evaluated. The rate of recurrent hemorrhage after fetal IPH is not known, and represents a critical gap in prognostication and care.

## Conclusions

Fetal intracerebral hemorrhage is increasingly recognized, however, the available literature to date is limited by small studies, an incomplete understanding of underlying etiologies, and limited data regarding pregnancy, infant, and childhood outcomes. Given that many pregnancies of fetal ICH end in the context of fetal demise or pregnancy termination, additional research to understand factors that influence pregnancy outcomes are critically needed. Current data suggest favorable neurodevelopmental outcomes in isolated low-grade GMH-IVH, whereas cases with parenchymal hemorrhage are reported to be associated with neuromotor impairments in the vast majority. Cognitive and epilepsy outcomes are not well described. Progression of post-hemorrhagic ventricular dilatation is associated with postnatal shunt placement, although rates of hydrocephalus vary markedly by study.

Systematic investigation for an etiology as recommended in these practical guidelines will enable a more standardized approach in clinical practice, which will inform understanding of the relative frequency of different etiologies by imaging pattern. Prospective large-scale studies are needed and the practical recommendations outlined in this review may serve as the foundation for a registry to capture etiology and outcomes across populations.
